# Structure of Gentlyase, the neutral metalloprotease of *Paenibacillus polymyxa*

**DOI:** 10.1107/S0907444912041169

**Published:** 2012-12-20

**Authors:** Armin Ruf, Martine Stihle, Jörg Benz, Manfred Schmidt, Harald Sobek

**Affiliations:** a pRED Pharma Research and Early Development, Small Molecule Research, Discovery Technologies, F. Hoffmann-La Roche Ltd, CH-4070 Basel, Switzerland; b Roche Diagnostics GmbH, Nonnenwald 2, 82377 Penzberg, Germany

**Keywords:** thermolysin, neutral proteases, calcium binding, Gentlyase

## Abstract

Gentlyase is a bacterial extracellular metalloprotease that is widely applied in cell culture and for tissue dissociation and that belongs to the family of thermolysin-like proteases. The structure of thermolysin has been known since 1972 and that of *Bacillus cereus* neutral protease since 1992. However, the structure determination of other *Bacillus* neutral proteases has been hindered by their tendency to cannibalistic autolysis. High calcium conditions that allow the concentration and crystallization of the active Gentlyase metalloprotease without autoproteolysis were identified using thermal fluorescent shift assays. X-ray structures of the protease were solved in the absence and in the presence of the inhibitor phosphoramidon at 1.59 and 1.76 Å resolution, respectively. No domain movement was observed upon inhibitor binding, although such movement is thought to be a general feature of the thermolysin-like protease family. Further analysis of the structure shows that the observed calcium dependency of Gentlyase stability may arise from a partly degenerated calcium site Ca1–2 and a deletion near site Ca3.

## Abstract 2

The crystal structure of the metalloprotease Gentlyase is described and compared with the structures of other related thermolysin-like proteases.

## 1. Introduction

The neutral metalloprotease thermolysin from *Bacillus thermoproteolyticus* was the first thermostable protein (*T*_50_ = 360.0 K) for which a structure was published (Matthews *et al.*, 1972 ▶). Owing to its availability, crystallizability and the quality and robustness of its crystals, thermolysin has often been used as a surrogate to model human metalloproteases for protease–inhibitor structures or for testing soaking methods (English *et al.*, 1999 ▶). It subsequently became one of the few proteins with more than 100 entries in the PDB (http://www.pdb.org) and lent its name to a whole family of neutral zinc metallo­proteases, which are also called thermolysin-like proteases (TLPs). At first sight it is therefore surprising that of the many other neutral proteases secreted by bacteria of the genus *Bacillus*, only the structure of *B. cereus* TLP has been deposited in the PDB (Stark *et al.*, 1992 ▶). However, their tendency to autoproteolysis (Veltman *et al.*, 1998 ▶) makes the isolation and crystallization of *Bacillus* neutral proteases that are less stable than thermolysin a difficult task.

TLPs consist of two domains that are flexibly connected by a central helix. The N-terminal domain contains several β-­strands and one α-helix, whereas the C-terminal domain is rich in α-helices. The active site is located between the two domains and the central α-helix carries some of the amino acids of the catalytic centre. The thermostable *Bacillus* neutral metalloproteases bind four calcium ions in addition to one catalytic Zn atom. Two Ca^2+^ ions are bound in one double calcium-binding site (Ca1–2) and two Ca^2+^ ions are bound in the single calcium-binding sites Ca3 and Ca4 (Stark *et al.*, 1992 ▶; Veltman *et al.*, 1998 ▶). The calcium-binding sites Ca1–2 and Ca4 are located in the C-terminal domain, whereas Ca3 is located in the N-terminal domain of the proteases. The single sites Ca3 and Ca4 are absent in thermolabile TLPs, suggesting that they are important determinants of stability (Eijsink *et al.*, 2011 ▶). In the TLP of *B. stearothermophilus* (TLP-ste), it has been shown that the Ca3 site is responsible for calcium-dependent stability in the 0.1–10 m*M* calcium concentration range. Formation of the Ca3 site is crucial for the structural integrity of this region, the local unfolding of which is the rate-limiting step in thermal inactivation by irreversible intermolecular proteolysis (Veltman *et al.*, 1998 ▶). The double calcium-binding site Ca1–2 is well conserved in the TLP family and inspection of structures of thermolysin from *B. thermo­proteolyticus* and TLP from *B. cereus* indicated that it would be impossible for the folded proteins to exist in the absence of at least Ca1 (Veltman *et al.*, 1998 ▶; Roche & Voordouw, 1978 ▶; Matthews *et al.*, 1972 ▶). Indeed, among these four Ca^2+^-binding sites Ca1 is the only one that is also present in all published structures of non-*Bacillus* TLPs such as aureolysin from *Staphylococcus aureus*, elastase from *Pseudomonas aerugin­osa* and MCP-02 from *Pseudoalteromonas* sp. (Banbula *et al.*, 1998 ▶; Thayer *et al.*, 1991 ▶; Gao *et al.*, 2010 ▶).

The TLP Gentlyase, the neutral metalloprotease from *Paenibacillus polymyxa* (EC 3.4.24.28), is an extracellular metalloprotease that cleaves fibronectin, collagen IV and, to a lesser extent, collagen and has maximum activity at neutral pH (Fogarty & Griffin, 1973 ▶; Griffin & Fogarty, 1973 ▶). This enzyme is used to gently disperse mammalian cells and tissues (Matsumura, Nitta *et al.*, 1975 ▶; Matsumura, Yamanaka *et al.*, 1975 ▶) and is widely applied in cell-culture procedures or for the isolation and tissue dissociation of primary cells. Its amino-acid sequence is highly similar to those of other neutral zinc metalloproteases (Stark *et al.*, 1992 ▶). Sequence analysis of the neutral metalloprotease from *P. polymyxa* shows that the enzyme has two amino-acid deletions (three and five amino acids) in the regions of the calcium-binding sites Ca3 and Ca1–­2, respectively, and it is therefore unknown whether these calcium-binding sites are retained. Similar to the TLP of *B. stearothermophilus*, Gentlyase shows autoproteolysis in the presence of low calcium concentrations (<5 m*M*) and auto­proteolysis is prevented in the presence of high concentrations of calcium (100 m*M*).

Here, we report (i) a method that can be generally applied to identify suitable conditions to keep active TLP stable for crystallization and (ii) the structure of Gentlyase, the TLP of *P. polymyxa*, at 1.59 Å resolution in the absence of and at 1.76 Å resolution in the presence of phosphoramidon, the natural TLP inhibitor from *Streptomyces tanashiensis*. The structures show the structural and functional consequences of the two deletions in the amino-acid sequence that are unique to the Gentlyase metalloprotease among *Bacillus* neutral proteases.

## 2. Methods

### 2.1. Protein production and characterization

Recombinant neutral protease from *P. polymyxa* was purified from the fermentation supernatant of *B. amylo­liquefaciens* cells. Following ammonium sulfate precipitation, the enzyme was purified to homogeneity using hydrophobic interaction chromatography. The purity of the enzyme preparations was analyzed by size-exclusion chromatography using a GE Superdex 75 (10/30) column equilibrated with 50 m*M* Tris–HCl, 300 m*M* NaCl, 100 m*M* CaCl_2_ pH 7.5. Protein concentrations were determined by measuring the optical density at 280 nm using a molar extinction coefficient of ∊_280 nm_ = 49 280 *M* ^−1^ cm^−1^. Protease activity was determined using a casein assay (Fujii *et al.*, 1983 ▶). To prevent autoproteolysis, the enzyme was stored in the presence of high calcium concentrations (50 m*M* HEPES, 1 *M* NaCl, 100 m*M* CaCl_2_ pH 8.2).

Calcium-depleted and high-calcium Gentlyase samples were prepared by buffer-exchange using a size-exclusion chromatography step. To completely avoid any autoproteolytic activity, samples of calcium-depleted and high-calcium Gentlyase were produced and analysed at low pH (pH 5.0) and in the presence of the inhibitor phosphoramidon. The proteolytic activity was inhibited by adding phosphoramidon (final concentration 0.61 m*M*) to enzyme samples (2 mg ml^−1^). Calcium-depleted Gentlyase was prepared by applying a 500 µl aliquot onto a Superdex 75 (10/30) column equilibrated with 50 m*M* MES buffer pH 5.0, 300 m*M* NaCl, 31 µ*M* phosphoramidon. High-calcium Gentlyase samples were prepared under identical conditions but with the buffer containing 100 m*M* CaCl_2_. Fractions from each chromatography were collected and enzyme pools were stored at 253 K. To detect residual autolytical activity, the enzyme pools were further analysed for degradation products on an analytical HLPC column (Tosoh TSK G3000SW equilibrated with 50 m*M* MES buffer pH 5.0, 300 m*M* NaCl, 31 µ*M* phosphoramidon and 0 or 100 m*M* CaCl_2_). To determine the thermal stability, samples were analysed in a thermal unfolding assay (Yeh *et al.*, 2006 ▶). The assays were performed using a LightCycler 480 instrument (Roche Applied Science). SYPRO Orange was obtained from Molecular Probes Inc. (Eugene, Oregon, USA) and was diluted 1:10 in DMSO. Protein samples (typically 6.5 µg) were in 50 m*M* MES buffer pH 5.0, 300 m*M* NaCl, 0.3 m*M* phosphoramidon and 0 or 100 m*M* CaCl_2_. SYPRO Orange was added at a 1:1430 dilution. The excitation wavelength was 483 nm and emission was measured at 568 nm. Assays were performed in a temperature range from 310 to 367 K with a temperature ramp of 3.6 K min^−1^.

### 2.2. Crystallization and structure determination

For crystallization trials, the protein was concentrated to 10 mg ml^−1^. Screening was performed in sitting drops at 277 K and crystals grew from several conditions containing PEG. A crystal grown from 0.2 *M* NaCl, 0.1 *M* HEPES pH 7.5, 25% PEG 3350 (Index condition F12) was soaked with the inhibitor phosphoramidon at 10 m*M* and a crystal grown from 0.2 *M* MgCl_2_, 0.1 *M* HEPES pH 7.5, 25% PEG 3350 (Index condition G12) was harvested directly. Both crystals were flash-cooled in liquid nitrogen without further addition of cryoprotectant. Data collection was performed at 100 K on beamline XS10A at the Swiss Light Source. The wavelength was 1 Å. Images were processed with *XDS* (Kabsch, 2010 ▶) and scaled with *SADABS* (Bruker AXS) or *XDS*. The structure was solved by molecular replacement with *Phaser* (McCoy *et al.*, 2007 ▶) using thermolysin (PDB entry 3fvp; L. Englert, A. Biela, M. Zayed, D. Hangauer, A. Heine & G. Klebe, unpublished work) as a search model. It was rebuilt with *ARP*/*wARP* (Perrakis *et al.*, 1999 ▶) and refined with *REFMAC* (Murshudov *et al.*, 2011 ▶); further structural modelling was performed with *Coot* (Emsley & Cowtan, 2004 ▶). The *CCP*4 program suite was used throughout (Winn *et al.*, 2011 ▶). The Ramachandran quality of the structural models was calculated by *Coot* to be 96.8/3.2/0.0 for the peptide complex and 93.3/6.5/0.2 for the phosphoramidon complex, where the values reflect the percentage of amino-acid residues in the core, allowed and disallowed regions of the Ramachandran plot, respectively. Data-collection and refinement statistics are given in Table 1 ▶.

## 3. Results and discussion

### 3.1. Protein stability

The stability of the metalloprotease of *P. polymyxa* is strongly dependent on the concentration of calcium (Fogarty & Griffin, 1973 ▶). In the presence of high calcium concentrations (100 m*M*) the half-life at pH 8.2 is about 7 d at room temperature, whereas at low temperatures (253 and 203 K) the enzyme is stable for months. In the absence of calcium, complete autolysis takes place within minutes (data not shown). This calcium-dependent stability is similar to that of TLP of *B. stearothermophilus*, for which a local unfolding process is considered to be the rate-determining step in heat inactivation owing to autolytic degradation (Veltman *et al.*, 1998 ▶). Earlier studies of TLP stability relied on thermal inactivation with enzymatic activity as readout (Veltman *et al.*, 1998 ▶; Van den Burg *et al.*, 1994 ▶). To detect the unfolding of Gentlyase metalloprotease independently of the overall thermal inactivation by autolytic degradation, we directly analyzed the thermal unfolding in the absence of the autolytic degradation process. To prevent autolysis, the phosphoramidon-inhibited protease complex was first prepared in both a calcium-depleted and a calcium-containing form and both were then analyzed in a thermal unfolding assay. The calcium-depleted inhibited metalloprotease showed a significantly reduced apparent melting temperature of 337 K compared with 347 K for the calcium-containing enzyme in 100 m*M* CaCl_2_ (Supplementary Fig. S1^1^). The lower thermal stability of the calcium-depleted Gentlyase metalloprotease indicates that this form is more easily unfolded and explains why it is more susceptible to thermal inactivation by autolysis than the calcium-containing form.

By using thermal fluorescent shift assays on the inhibited protease, the rate-limiting unfolding step was detected directly and independently of the overall thermal inactivation, which is the convoluted consequence of partial unfolding and autolysis.

### 3.2. Overall protein structure

**Figure 1:**
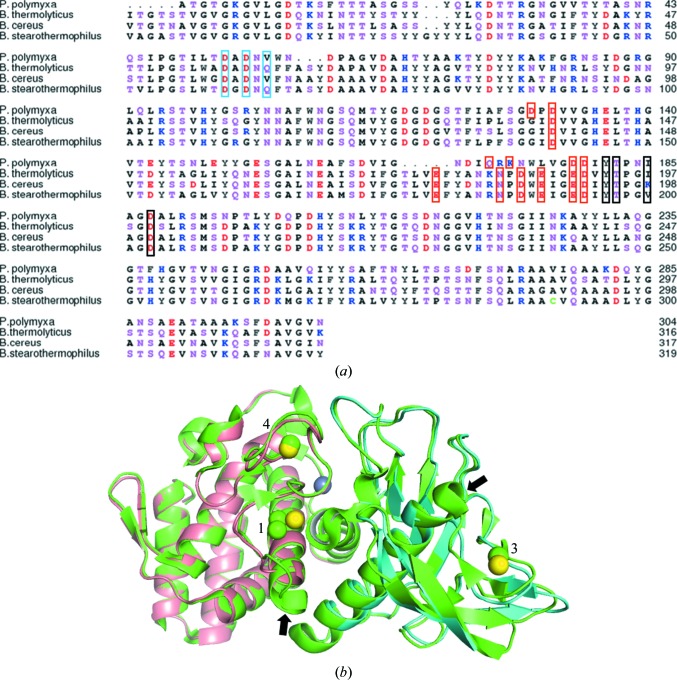
(*a*) Sequence alignment between the neutral proteases from *Paenibacillus polymyxa* (Gentlyase), *Bacillus thermoproteolyticus* (thermolysin), *B. cereus* (UniProt ID Q63G45_BACCZ) and *B. stearothermophilus* (NPRT_GEOSE). The numbering shown corresponds to the mature proteases. Residues liganding Ca^2+^ in Ca1–2, Ca3 and Ca4 are boxed in red, blue and black, respectively. Acidic, basic, polar and nonpolar residues are coloured red, blue, magenta and black, respectively. (*b*) Superposition of the overall structure of *P. polymyxa* neutral protease (N-terminal domain in cyan and C-terminal domain in blue) with that of thermolysin from *B. thermoproteolyticus* (green; PDB entry 3fvp). Calcium-binding sites Ca1–­2, Ca3 and Ca4 are discernible by the yellow Ca^2+^ ions and are labelled 1, 3 and 4, respectively. The active site is marked by the grey Zn^2+^ ion. There is overall high structural similarity, except for the two helices close to the calcium-binding sites Ca1–2 and Ca3, which are shortened by one turn in *P. polymyxa* neutral protease (black arrows) owing to the two sequence deletions.

The *P. polymyxa* neutral protease (Gentlyase) has 56% sequence identity to thermolysin and has two short deletions corresponding to thermolysin residues 64–66 and 174–178 (Fig. 1 ▶ *a*). The Gentlyase metalloprotease structure shows the overall architecture of a typical TLP. The N-terminal domain contains a series of β-strands that cradle one long α-helix. The C-terminal domain is mainly α-helical and carries the terminal four-helix bundle. The C-terminal and N-terminal domains are connected by a central α-helix containing several catalytically crucial residues. The two sequence deletions in the Gentlyase metalloprotease lead to the loss of the N-terminal turn of the long helix in the N-terminal domain near Ca3 and to a similar C-­terminal shortening of the helix in the C-terminal domain near Ca1–2 (Fig. 1 ▶ *b*). The backbone r.m.s.d. to the best resolved thermolysin structure (PDB entry 3fvp) is 0.59/0.61 Å over 302 residues and the backbone r.m.s.d. to *B. cereus* TLP (PDB entry 1npc; Stark *et al.*, 1992 ▶) is 0.94/1.01 Å, which is similar to the r.m.s.d. of 0.99 Å over 316 residues between thermolysin and *B. cereus* TLP and would be expected from the high sequence similarity. The backbone r.m.s.d.s between the isolated Gentlyase N-terminal or C-terminal domains and thermolysin alone are 0.54 Å (122 residues) and 0.58 Å (127 residues), respectively, indicating identical domain arrangement in the thermolysin and Gentlyase structures.

### 3.3. Active site and inhibitor binding

*B. cereus* TLP displays domain movement upon inhibitor binding (Holland *et al.*, 1992 ▶), whereas this was not observed for thermolysin and aureolysin from *S. aureus* (Banbula *et al.*, 1998 ▶). Sadly, the pair of Gentlyase structures described here do not help in clarifying the question of whether the domain movement upon inhibitor binding is a general feature of all *Bacillus* TLPs. The lack of domain movement observed for the Gentlyase metalloprotease can be explained by the dipeptide fortuitously bound to the active site in the uninhibited form. The same rationale was applied to explain the lack of movement originally observed for thermolysin, in which the un­inhibited crystals also showed electron density for a dipeptide. In thermolysin this density was interpreted as the terminal Val-Lys of thermolysin, because this ‘dipeptide might have been cleaved from the intact protein during protein purification or during crystallization’ (Holland *et al.*, 1992 ▶). In the uninhibited Gentlyase crystal the observed electron density for a dipeptide does not perfectly fit the side chains of any canonical amino acids. We used a Thr-Lys dipeptide model for refinement, which explains the electron density less poorly than Val-Lys. However, the observed electron density is likely to represent a mixture of proteolysis and autolysis products. Another possible explanation for the lack of domain movement observed in the Gentlyase structure is the loss of the double glycine motif that precedes the central helix and is close to the thermolysin-like protease hinge-bending axis reported by Holland *et al.* (1992 ▶). Thermolysin Gly135-Gly136 and *B. cereus* TLP Gly136-Gly137 correspond to Gly128-Asp129 in the Gentlyase metalloprotease.

**Figure 2:**
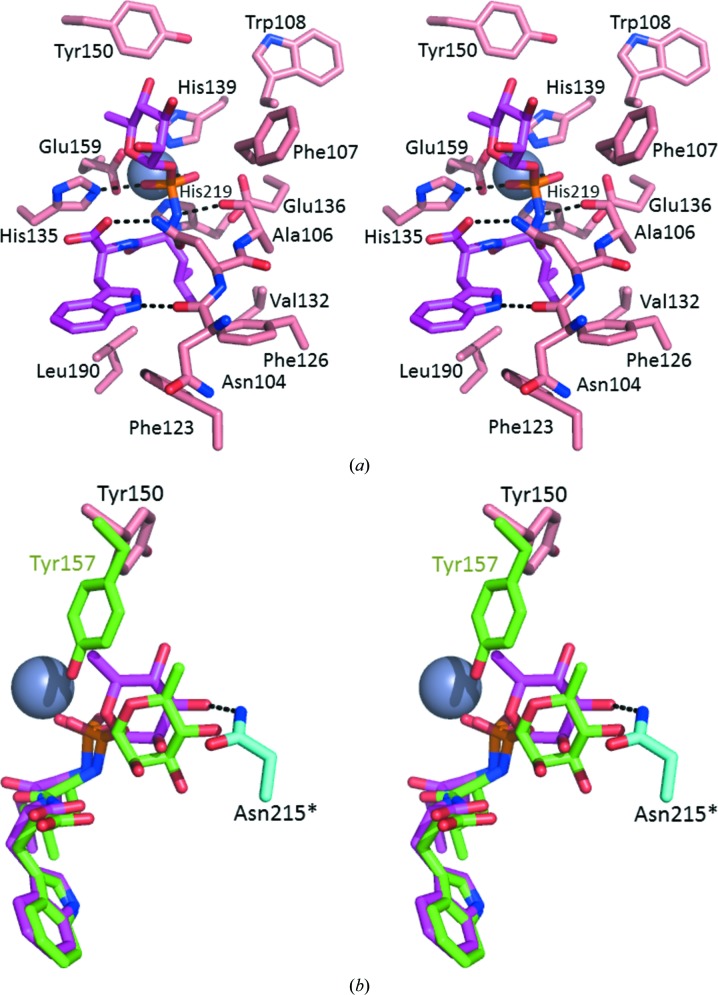
(*a*) Close-up stereoview of the active site with the inhibitor phosphoramidon (magenta) that was soaked into the crystals. It displays the typical binding mode of phosphoramidates to TLP. (*b*) Superposition of phosphoramidon bound to the neutral proteases Gentlyase (pink) and thermolysin (green) shows the overall good agreement of the Trp, Leu and phosphoramidate moieties of the inhibitors. In the Gentlyase crystal a different conformation of the rhamnose moiety is observed that forms a hydrogen bond across a crystal lattice contact to Asn215 of another protein chain (cyan) and displaces the Tyr150 side chain.

The residues lining the active sites are identical in thermolysin and Gentlyase metalloproteases; indeed, the Gentlyase phosphoramidon binding is similar to the thermolysin phosphoramidon binding in PDB entry 1tlp, in which Zn^2+^, the His219 imidazole, the Glu136 carboxyl and the Ala106 carbonyl coordinate the phosphoramidate (Tronrud *et al.*, 1986 ▶). The interactions of the two proteases with the phosphoramidate, Leu and Trp moieties of phosphoramidon are almost identical within co­ordinate error (Fig. 2 ▶ *a*). The only significant difference from the thermolysin phosphoramidon binding is observed in the rhamnose group of the inhibitor. Surprisingly, the Gentlyase Tyr150 side-chain conformation differs from that of thermolysin Tyr157, allowing the rhamnose moiety of the phosphoramidon to bind in an orientation rotated by approximately 60° around the glycosidic bond compared with that in thermolysin, where this conformation would create unfavourable contacts of the rhamnose methyl group (Fig. 2 ▶ *b*). The same Tyr150 position was observed as an alternative conformation in our structure of the uninhibited Gentlyase metalloprotease, indicating its possible physiological relevance. The same phosphoramidon conformation is also observed in both independent copies in the Gentlyase crystal. However, the observed rhamnose conformation is also influenced by crystallization, because the rhamnose groups of both phosphoramidons in the crystallo­graphic asymmetric unit are involved in different crystal lattice contacts. In one instance the phosphoramidon hydroxy group O4 hydrogen bonds to Tyr151 and in the second instance hydroxy group O3 hydrogen bonds to Asn221 of a different crystal lattice neighbour. Independent corroborating evidence for the physiological relevance of the phospharamidon binding mode observed in Gentlyase crystals comes from the PDB: all other deposited phosphoramidon complexes, namely those of human neutral endopeptidase (Oefner *et al.*, 2000 ▶), *Pseudomonas aeruginosa* elastase (PDB entry 3dbk; D. B. McKay & M. T. Oovergard, unpublished work), endothelin-converting enzyme I (Schulz *et al.*, 2009 ▶) and *Mycobacterium tuberculosis* zinc metalloprotease ZMP1 (Ferraris *et al.*, 2011 ▶) display this rhamnose orientation. Moreover, Tyr155 in phosphoramidon-inhibited *P. aeruginosa* elastase also differs in side-chain conformation from thermolysin Tyr157.

### 3.4. Calcium-binding sites

Only calcium site Ca4, which consists of Tyr181, Thr182, Ile185 and Asp188, is conserved from its counterpart in thermolysin. The other Ca sites differ significantly and may explain the different calcium dependency of Gentlyase stability.

#### 3.4.1. Calcium site Ca1–2

The structures of the neutral metalloprotease (NP) from *B. cereus* (Stark *et al.*, 1992 ▶) and of thermo­lysin from *B. thermoproteolyticus* (Matthews *et al.*, 1972 ▶) both show two calcium ions bound in a double calcium-binding site Ca1–2. In thermolysin the calcium ions are liganded by residues Asp138, Glu177, Asn183, Asp185, Glu188 and Glu190. There is a five-amino-acid deletion in the corresponding region of the Gentlyase metalloprotease from *P. polymyxa* (corresponding to thermolysin residues 174–178), which results in a drastic change in this calcium-binding site (Figs. 1 ▶ *a* and 3 ▶). Here, the Ca1–2 site lacks the Asp185 side chain that coordinates both Ca^2+^ ions in thermolysin. Therefore, a single calcium site is formed. Interestingly, the amino group of Lys172 in Gentlyase is located very close to the former Ca2 site and its positive charge may partially compensate for the missing charge of the second Ca^2+^. Also, in *P. aeruginosa* elastase, the only other TLP of known structure, which has a single Ca1 site instead of the double Ca1–2 site, there are two positively charged side chains, Arg179 and Lys181, in close proximity to the Ca2 region (Thayer *et al.*, 1991 ▶).

#### 3.4.2. Calcium site Ca3

**Figure 4:**
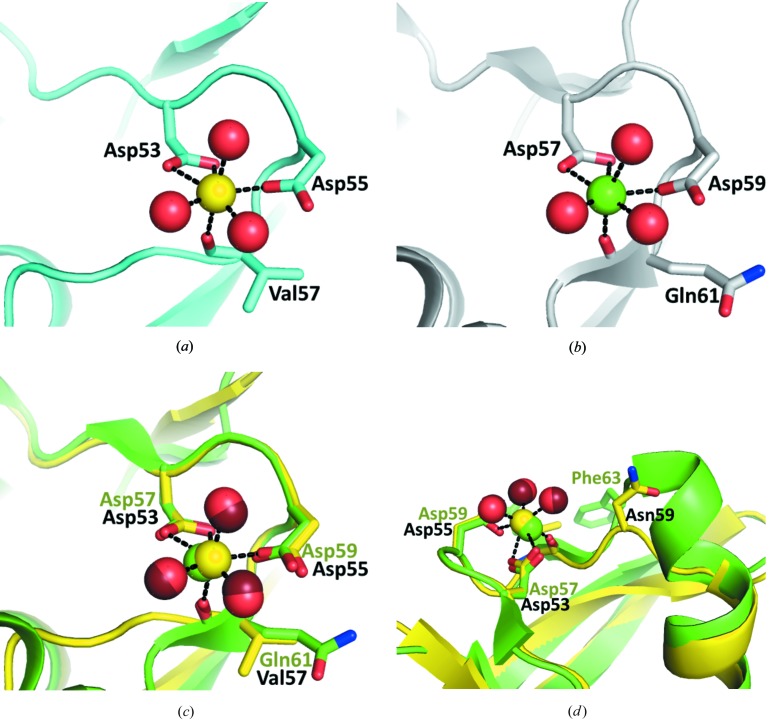
(*a*) The Ca^2+^-binding site Ca3 in the N-terminal domain of the *P. polymyxa* neutral protease Gentlyase. Water molecules coordinating the Ca^2+^ ion (yellow) are shown as red spheres. (*b*) The Ca3 site in the prototypic neutral protease thermolysin from *B. thermoproteolyticus* (PDB entry 3fvp). (*c*, *d*) Superimposition of the neutral protease Gentlyase (yellow) with thermolysin (green) shows that the deletion in Gentlyase does not affect the Ca3 site. However, the backbone conformation and side-chain orientation of Asn59 adjacent to the deletion is affected. The corresponding Phe63 is reported to confer thermostability on thermolysin or *B. stearothermophilus* neutral protease by stabilizing the N-terminal β-sheet through hydrophobic interactions with the side chain (Van den Burg *et al.*, 1994 ▶). Owing to the three-residue deletion after Asn59 these stabilizing hydrophobic contacts cannot be formed in Gentlyase, even if the thermolysin Phe63 were not changed to Asn59 in Gentlyase.

Sequence alignment of *Bacillus* TLP shows a deletion close to Ca3 that is unique to the Gentlyase metalloprotease (Fig. 1 ▶ *a*). Because mutations in Ca3 have been shown to affect the calcium-dependence of the stability of *B. stearothermophilus* TLP by a mechanism that involves local unfolding around Ca3 (Veltman *et al.*, 1998 ▶; Eijsink *et al.*, 1995 ▶), it was tempting to speculate that structural differences at Ca3 are responsible for the marked Ca^2+^ dependence of Gentlyase stability. However, the structural superimposition of *P. polymyxa* neutral protease with thermolysin of *B. thermoproteolyticus* shows that the deletion of thermolysin residues 64–66 in *P. polymyxa* neutral protease does not affect Ca3 formed by residues Asp53, Asp55 and Val57 in the vicinity (Fig. 4 ▶).

In thermolysin, Phe63, which is located on the surface near Ca3, stabilizes the N-terminal β-sheet through hydrophobic interactions with amino acids (Val9, Gln17, Arg11 and Gln61) of the three strands in this β-sheet (Eijsink *et al.*, 1995 ▶, 2011 ▶). The contribution of Phe63 to the stability of the enzyme has been demonstrated for *B. stearothermophilus* TLP, in which the introduction of arginine, lysine or bulky hydrophobic amino acids increased the thermal stability significantly, whereas the introduction of hydrophilic residues reduced the thermal stability (Van den Burg *et al.*, 1994 ▶). Phe63 in thermolysin aligns with Asn59 in Gentlyase. However, owing to the neighbouring C-terminal deletion of three residues (thermolysin residues 64–66), the conformation of Asn59 differs from that of thermolysin Phe63 and the Asn side chain points in the opposite direction away from the N-terminal β-sheet (Fig. 4 ▶ *d*). Consequently, the stabilizing hydrophobic contacts between Phe63 and the β-sheet cannot be formed in Gentlyase. However, thermolysin residues Val7 and Val9, the side chains of which constitute most of the hydrophobic patch on the N-terminal β-sheet interacting with Phe59, are replaced by threonines in Gentlyase, which do not create an un­favourable hydrophobic surface patch in the absence of Phe59.

## 4. Conclusion

In summary, we have directly shown the pronounced structural stabilization at high calcium concentrations of Gentlyase, the neutral metalloprotease of *P. polymyxa*, which is widely used in cell culture and tissue dissociation. By using thermal fluorescent shift assays on the inhibited protease, which directly detected the rate-limiting unfolding step independently of the overall thermal inactivation, we identified suitable high-calcium conditions that allowed crystallization of the active thermolysin-like protease. We then solved the structures of the Gentlyase metalloprotease both in complex with the inhibitor phosphoramidon and without inhibitor. Because no domain movement was observed upon inhibitor binding, as for thermolysin and for aureolysin, the domain movement upon inhibitor binding observed in *B. cereus* TLP could not be confirmed as a general feature of TLPs. Comparison with other TLPs showed that the marked calcium dependency of Gentlyase stability may arise from a partly degenerated calcium site Ca1–2 and a deletion near site Ca3.

## Supplementary Material

PDB reference: Gentlyase, peptide complex, 4ger

PDB reference: phosphoramidon complex, 4b52

Supplementary material file. DOI: 10.1107/S0907444912041169/dw5030sup1.pdf

## Figures and Tables

**Figure 3:**
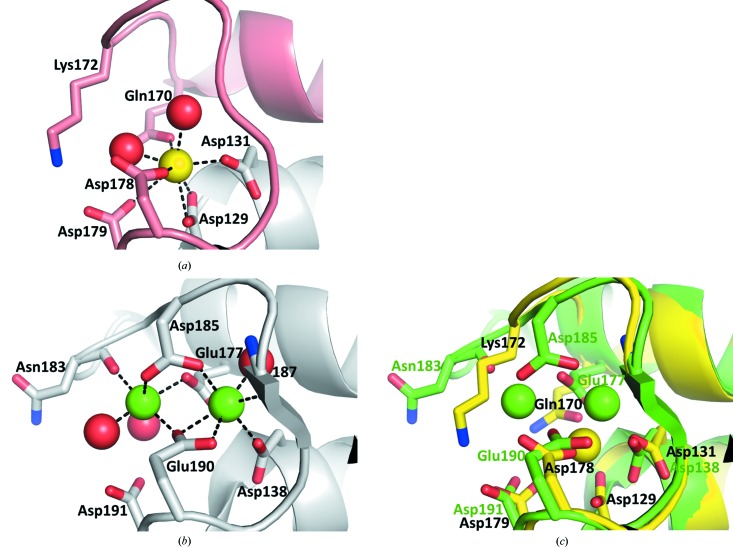
The Ca^2+^-binding site Ca1–2 is degenerated in the C-terminal domain of the *P. polymyxa* neutral protease Gentlyase. (*a*) The double calcium site Ca1–2 is occupied by only one Ca^2+^ in both structures of *P. polymyxa* neutral protease. Water molecules coordinating the Ca^2+^ ion (yellow) are shown as red spheres. (*b*) Ca1–2 in the prototypic neutral protease thermolysin from *B. thermoproteolyticus* (PDB entry 3fvp). (*c*) Superimposition of Ca1–2 in *P. polymyxa* neutral protease (yellow) with that of thermolysin from *B. thermoproteolyticus* (green) shows that the Asp185 coordinating the first Ca^2+^ in thermolysin is substituted by two water molecules and that Lys172 provides the missing positive charge of the second Ca^2+^ in *P. polymyxa* neutral protease. Waters were omitted for clarity.

**Table 1:** Data-collection and refinement statistics Each data set was collected from one single crystal. Values in parentheses are for the highest resolution shell.

	Peptide complex (PDB entry 4ger)	Phosphoramidon complex (PDB entry 4b52)
Data collection		
Space group	*P*2_1_	*P*2_1_
Unit-cell parameters		
*a* (Å)	61.0	61.1
*b* (Å)	77.1	77.0
*c* (Å)	64.1	64.4
α = γ (°)	90.0	90.0
β (°)	103.2	103.6
Resolution (Å)	48.9–1.59 (1.69–1.59)	48.6–1.76 (1.86–1.76)
*R*_merge_	0.141 (0.661)	0.149 (0.629)
〈*I*/σ(*I*)〉	7.4 (1.3)	7.9 (1.6)
Completeness (%)	97.6 (96.2)	97.8 (97.7)
Multiplicity	3.4 (3.3)	3.3 (3.3)
Refinement		
Resolution (Å)	48.9–1.59 (1.63–1.59)	48.6–1.76 (1.81–1.76)
No. of reflections	68487 (4527)	53023 (3542)
*R*_cryst_/*R*_free_ (%)	20.2/24.3 (25.7/41.1)	19.8/24.8 (31.0/35.8)
No. of atoms		
Total	5275	5295
Protein	4588	4622
Ligand/ion	76	83
Water	611	590
*B* factor (Å^2^)	14.6	16.5
R.m.s. deviations		
Bond lengths (Å)	0.022	0.024
Bond angles (°)	2.052	2.136

## Footnotes

1 Supplementary material has been deposited in the IUCr electronic archive (Reference: DW5030). Services for accessing this material are described at the back of the journal.
