# Supplementary material for: Structure of Gentlyase, the neutral metalloprotease of Paenibacillus polymyxa
Source: Acta Crystallogr D Biol Crystallogr. 2012 Dec 20;69(Pt 1):24–31. doi: 10.1107/S0907444912041169 (PMC3532130; doi:10.1107/S0907444912041169)
Supplement: Supplementary file 1 [file d-69-00024-sup1.pdf]

Supplementary Material

Journal: Acta Crystallographica Section D: Biological Crystallography

Authors: Armin Ruf*, Martine Stihle, Jorg Benz, Manfred Schmidt and Harald Sobek

Title: Structure of Gentlyase, the neutral metalloprotease of Paenibacillus polymyxa

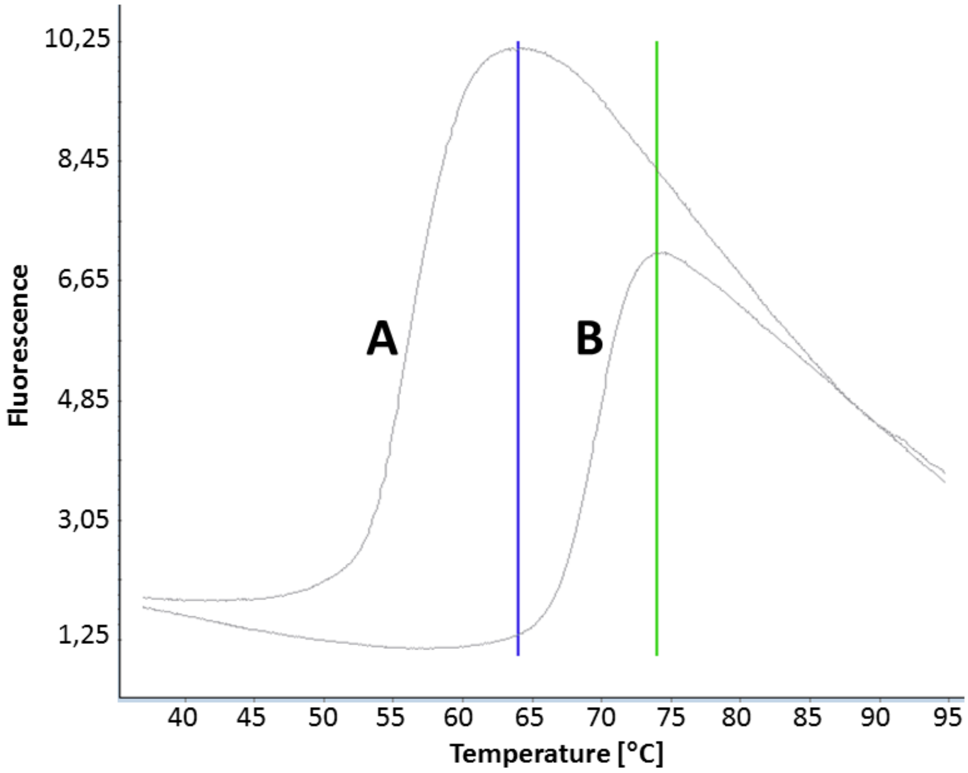

Supplementary Figure S1 Melting curves of calcium-depleted and calcium-containing forms of Gentlyase. 6.5 µg of calcium-depleted (curve A) and calcium-containing (curve B) form of Gentlyase were analysed in 50 mM MES buffer (pH 5.0), 300 mM NaCl, 0.3 mM phosphoramidon and 0 (curve A) or 100 mM (curve B) CaCl2.
